# Pleiotropic Effects of Isoflavones in Inflammation and Chronic Degenerative Diseases

**DOI:** 10.3390/ijms22115656

**Published:** 2021-05-26

**Authors:** Jurga Bernatoniene, Jurga Andreja Kazlauskaite, Dalia Marija Kopustinskiene

**Affiliations:** 1 Department of Drug Technology and Social Pharmacy, Faculty of Pharmacy, Medical Academy, Lithuanian University of Health Sciences, Sukileliu pr. 13, LT-50161 Kaunas, Lithuania; 2 Institute of Pharmaceutical Technologies, Faculty of Pharmacy, Medical Academy, Lithuanian University of Health Sciences, Sukileliu pr. 13, LT-50161 Kaunas, Lithuania; jurga.andreja.kazlauskaite@stud.lsmu.lt (J.A.K.); DaliaMarija.Kopustinskiene@lsmuni.lt (D.M.K.)

**Keywords:** isoflavones, genistein, daidzein, inflammation, cancer, degenerative diseases

## Abstract

Isoflavones are phytoestrogens of plant origin, mostly found in the members of the Fabaceae family, that exert beneficial effects in various degenerative disorders. Having high similarity to 17-*β*-estradiol, isoflavones can bind estrogen receptors, scavenge reactive oxygen species, activate various cellular signal transduction pathways and modulate growth and transcription factors, activities of enzymes, cytokines, and genes regulating cell proliferation and apoptosis. Due to their pleiotropic activities isoflavones might be considered as a natural alternative for the treatment of estrogen decrease-related conditions during menopause. This review will focus on the effects of isoflavones on inflammation and chronic degenerative diseases including cancer, metabolic, cardiovascular, neurodegenerative diseases, rheumatoid arthritis and adverse postmenopausal symptoms.

## 1. Introduction

Isoflavones are polyphenolic plant-derived compounds acting as phytoestrogens due to their structural similarity to 17-*β*-estradiol [1]. They are found as secondary plant metabolites in a conjugated form mainly in the members of the Fabaceae family, such as soybeans (*Glycine max* (L.) Merr.), red clover (*Trifolium pratense* L.), white clover (*Trifolium repens* L.), alfalfa (*Medicago sativa* L.), various lupin (*Lupinus*) species and beans (*Phaseolus vulgaris* L.). Depending on the endocrine levels of estrogen in an organism, isoflavones can act as estrogen agonists (at low concentrations) or antagonists (at high concentrations) [2,3,4]. Isoflavones can interact with both estrogen receptors *α* and *β*, with a much higher affinity for the latter [5], and mimic the action of estrogens on target organs, thereby exerting many health benefits when used in some hormone-dependent diseases [3,5,6,7,8].

**Figure 1:**
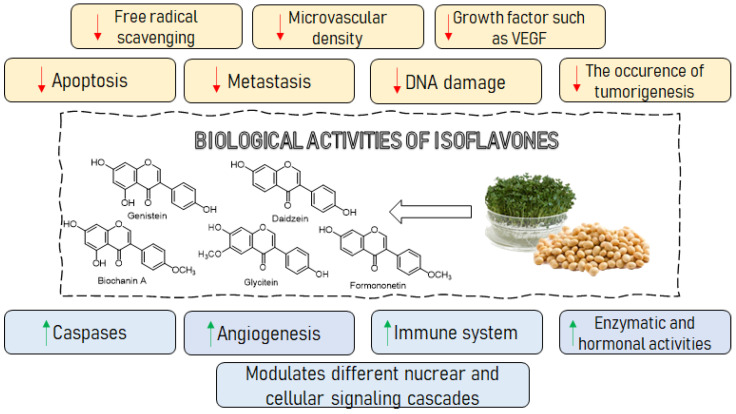
Biological activity of isoflavones. VEGF-vascular endothelial growth factor.

Isoflavones can alleviate many pathological conditions including cancer, metabolic, cardiovascular, neurodegenerative diseases, rheumatoid arthritis and adverse menopause symptoms (Figure 1). Recently, various aspects of isoflavones have been overviewed in detail in the following reviews [3,9,10,11,12,13]. Two mechanisms are implicated to be responsible for the beneficial effects of isoflavones—the estrogen receptor-mediated signaling pathway [2,7,8] and the modulation of other intracellular signaling pathways, e.g., phospholipase C, protein tyrosine kinase and mitogen-activated protein kinase [3,7,14,15].

## 2. Chemical Properties and Bioavailability of Isoflavones

**Figure 2:**
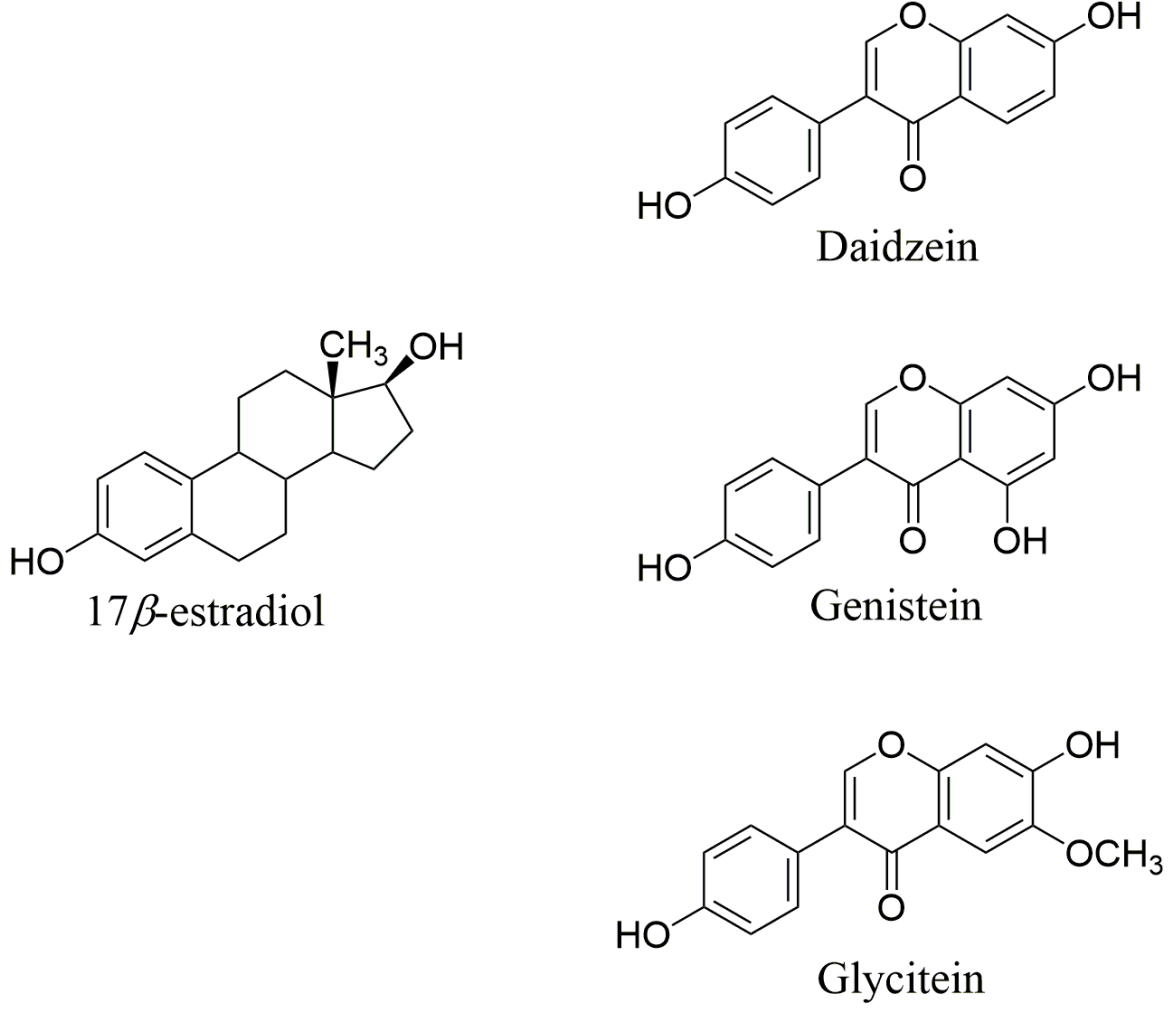
Main isoflavones and their structural similarity to 17*β*-estradiol.

Isoflavones form a group of distinct secondary metabolites produced predominately in leguminous plants. These secondary metabolites are formed by symbiotic relationship with the *Rhizobia* bacteria and the defense responses of leguminous plant [16]. Isoflavones are synthesized as part of the phenylpropanoid pathway, the same biosynthetic pathway of flavonoid biosynthesis [17]. The structures of the main isoflavones are presented in Figure 2.

**Figure 3:**
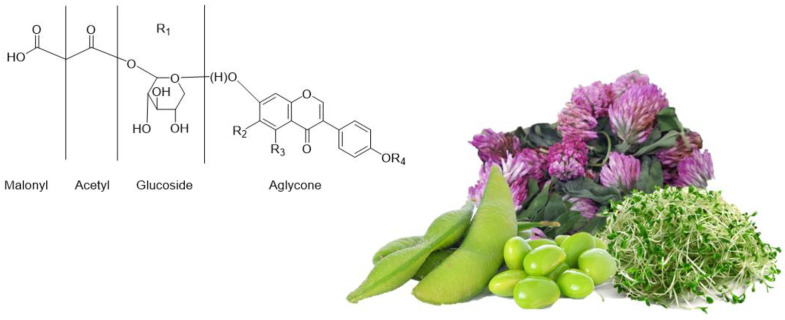
Chemical structure of isoflavones from Fabaceae family plants (alfalfa, red clover and soy).

Isoflavones contain 12 different isoforms that are divided into four chemical forms (Figure 3 and Table 1): aglycones, 7-*O*-glucosides, 6″-*O*-acetyl-7-*O*-glucosides and 6″-*O*-malonyl-7-*O*-glucosides [18]. It is also possible for any conjugated isoflavone to generate the aglycone form by cleavage of the glycosidic bond.

Some glycosides, including malonyl- and acetyl-isoflavones, are particularly unstable (Table 1). The use of drastic temperatures, pressure conditions and long extraction times may cause the degradation of isoflavonoids conjugates, changing the isoflavone profile. In addition, chemical hydrolysis leads to a marked increase in the concentration of aglycones present in the sample at the expense of the glucosides and hence augment the available amount of aglycones to be extracted [19,20].

Isoflavones in glycoside form are poorly absorbed in the small intestine, due to their higher molecular weight and hydrophilicity. However, gut microflora plays an important role in the bioconversion of isoflavones. Bacteria, mainly *Bifidobacterium* and *Lactobacillus* strains present in the gastrointestinal tract hydrolyze isoflavones to their corresponding bioactive aglycone forms [21,22]. Once hydrolyzed, aglycone forms are absorbed in the upper gastrointestinal tract by a passive diffusion [21].

Genistein and daidzein (aglycones) can be produced from their glucosides or from the precursors biochanin A and formononetin by intestinal *β*-glucosidase, these compounds are extensively metabolized in the intestine and liver [23]. After ingestion and hydrolysis, aglycones are absorbed in the small intestine completely in part or further metabolized into other metabolites (sulfonic or glucuronic acid conjugates) during demethylation and reduction reactions [24,25]. Along with bacterial metabolism, isoflavones are metabolized by phase-I and II isoenzymes in liver. Aglycones daidzein and genistein undergo hydroxylation catalyzed by Phase-I enzymes (cyptochrome P450) and glycitein is metabolized to mono- or di-hydrozylated glycitein metabolites [26].

Nevertheless, pharmacokinetic studies confirm that healthy adults absorb isoflavones rapidly and efficiently. The average time to ingested aglycones reach peak plasma concentrations in about 4–7 h, which is delayed to 8–11 h for the corresponding *β*-glycosides. Despite the fast absorption, isoflavones or their metabolites are also rapidly excreted [21]. The metabolites of daidzein found in human urine after soy supplementation are equol, O-desmethylangolensin, dihydrodaidzein and 4′,7-dihydroxyisoflavan-4-ol. Genistein is metabolized to dihydrogenistein and 2′,4′,6′,4″-tetrahydroxy-α-methyldeoxybenzoin. While equol and O-desmethylangolensin are considered as end products of the metabolism of daidzein, the metabolism of genistein has been shown to proceed to 2-(4-hydroxyphenyl)-propanoic acid and trihydroxybenzene by C-ring fission. The glycitein is metabolized to dihydroglycitein, 2′,4′,4″-trihydroxy-5′-methoxy-α-methyldeoxybenzoin and 6′-methoxy-4′,7-dihydroxyisoflavan. These compounds levels found in urine samples are much lower than genistein and daidzein metabolites, but still suggests that glycitein is converted to reduced metabolites by gut microflora [25].

Across the results of different studies there remain some inconsistencies regarding the factors that affect isoflavone bioavailability in humans, mainly due to the use of different study designs and diverse food sources of isoflavones in intra- and inter-studies [27]. For example, it was reported that fermented soy foods may enhance the absorption of isoflavones among the people who consume fermented soybean compared to those consuming non fermented soybean. It was explained that probiotic effects of fermented foods may result in an increase in the gut bacterial population [22].

Although isoflavones are potential endocrine disrupters and become cytotoxic at high doses [28], at physiological concentrations they are safe to use, only mild adverse gastrointestinal effects have been reported [11].

## 3. The Effects of Isoflavones in Inflammation

Inflammation is a rapid biological response of body tissues to harmful stimuli it is also known to be involved in a lot of diseases: obesity, atherosclerosis, rheumatoid arthritis, and even cancer [29]. Inflammation increases the vascular permeability resulting in the leukocyte migration into the injured tissues. The inflammatory mediators like tumor necrosis factor (TNF)-*α*, interferon (IFN)-*γ*, interleukins (IL) as well as chemokines play an important role in inflammation [30]. Steroidal or non-steroidal anti-inflammatory drugs are currently used for the inflammation treatment, but occasionally these drugs are accompanied with side effects, and also, they are not considered as a good clinical choice for the treatment of the chronic inflammatory disorders [19].

In alternative medicine crude plant extracts are used for the treatment of a wide variety of disorders including acute and chronic inflammation [31]. Recent investigations have demonstrated that the active constituents of these extracts exhibit not only anticancer, antimicrobial, and antiviral effects but also anti-inflammatory activity both in vitro and in vivo [32,33,34].

**Figure 4:**
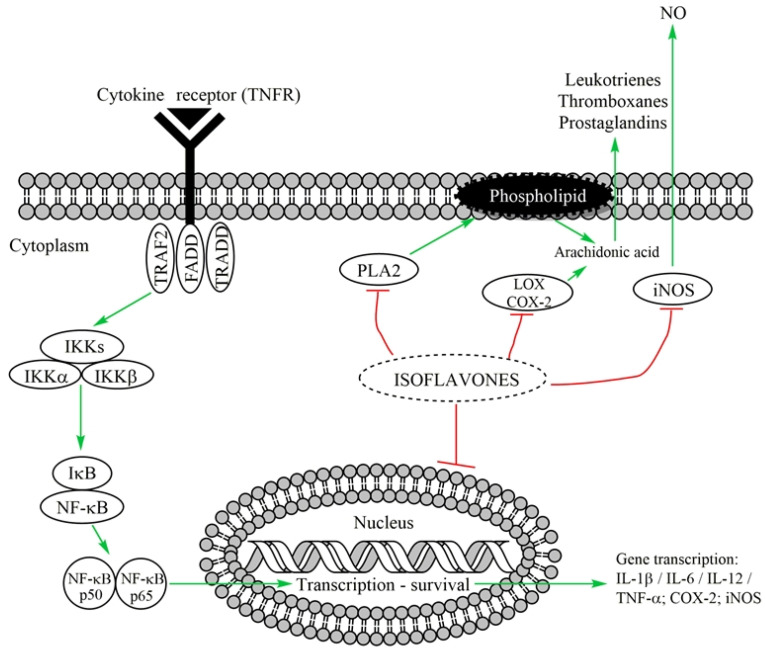
The effects of isoflavones in inflammation. TRAF2—tumor necrosis factor receptor associated factor-2, FADD—Fas-associated death domain protein, TRADD—TNFR1-associated death domain protein, IκB—inhibitory factor kappa B, IKK—IκB kinase, NF-κB—nuclear factor of kappa light polypeptide gene enhancer in B-cells, NO—nitric oxide, PLA2—phospholipase A2, LOX—lipoxygenase, COX-2—cyclooxygenase-2, iNOS—inducible nitric oxide synthase, IL—interleukin, TNF-α—tumor necrosis factor alpha. Green arrow—activation, up-regulation; red arrow—blocking, down-regulation.

It was speculated that isoflavones may act as anti-inflammatory agents because they can down-regulate cytokine-induced signal transduction [35] (Figure 4).

In a study by Chacko et al., it was reported that anti-inflammatory activity exerted by the isoflavone genistein involved inhibition of monocyte adhesion to cytokine-activated endothelial cells. This antiadhesive effect of genistein was dependent on the flow and was mediated via activation of peroxisome proliferator-activated receptor gamma (PPAR-*γ*) [33]. In recent years, an increasing number of investigations have consistently proven that isoflavones exhibit anti-inflammatory function [33]. The studies demonstrated that the specific isoflavones appeared to exhibit different effects on inflammatory processes. For example, IFN-*γ* induced signal transducer and activator of transcription 1 (STAT1) phosphorylation was reduced in human epithelial colorectal adenocarcinoma cells upon treatment with genistein [36]. Similarly, Jantaratnotai et al. concluded that genistein and daidzein possessed anti-inflammatory effects against lipopolysaccharide-activated microglia. These effects were mediated through inhibition of inducible nitric oxide synthase (iNOS) expression via the transcription factors, interferon regulatory factor-1 and phosphorylated STAT1 as well as a reduction in monocyte chemoattractant protein-1 (MCP-1) and IL-6 expression [37]. It was determined by Gredel et al. that isoflavone metabolites like equol can downregulate inflammatory cytokine production (IL-6, IL-8, TNF-α, IL-12) in several different immune cell subtypes [38].

In animal trials with isoflavones the potential therapeutic properties of isoflavones against *D*-galactosamine-induced inflammation and hepatotoxicity has been evaluated [39]. Isoflavones reduced the levels of nitric oxide (NO) and prostaglandin E2 (PGE_2_), and suppressed the production of *D*-galactosamine-induced proinflammatory cytokines, including TNF-*α* and IL-1*β* in male Wistar rats [39].

In human trials, 32 healthy and non-obese postmenopausal women without hormone therapy were randomly assigned to exercise and placebo or exercise and isoflavone supplementation (100 mg) groups [40]. Blood samples were analyzed for the lipid profile, interleukin-6, interleukin-8, superoxide dismutase, total antioxidant capacity, and thiobarbituric acid reactive substances. The results of the study showed that isoflavones did not promote additive or independent effects on the lipid profile and on inflammatory and oxidative stress markers in non-obese postmenopausal women, but the intake of the isoflavones was relatively low and the research was too short for detecting the effects of isoflavone supplementation associated with the combined exercise [40]. In other study, with obese and overweighed woman, where 34 were assigned to exercise and placebo or exercise and isoflavones groups, the results were more promising [41]. The results showed an increase in TNF-*α*, but isoflavones enhanced the beneficial effects of mixed-exercise training on body composition and C-reactive protein in overweight or obese postmenopausal women [41]. When the subjects received isoflavone-containing soy-based nutritional supplements (soy group) or isoflavone-free milk protein (control group) for 8 weeks, isoflavone-rich diet reduced the markers of inflammation (C-reactive protein, IL-6 and TNF-*α*) in the soy group [42].

Thus, isoflavones could act as anti-inflammatory agents in various *in vitro* and *in vivo* models of inflammation.

## 4. The Role of Isoflavones in Chronic Degenerative Diseases

Genistein, daidzein, and glycitein are the three most bioavailable isoflavones and there is a growing evidence in their protective effects in alleviating chronic-degenerative diseases [43]. The main molecular targets of isoflavones include caspases, B-cell lymphoma 2 (Bcl-2) protein, Bcl-2-associated X protein, nuclear factor-κB (NF-κB), various components of signal transduction pathways, e.g., phosphoinositide 3-kinase/Akt, extracellular signal-regulated kinase (ERK)1/2, mitogen-activated protein kinase (MAPK) and Wnt/*β*-catenin [44].

### 4.1. Effects of Isoflavones in Cancer

In the past two decades isoflavones have been intensively studied due to their potential beneficial effects in cancer. Isoflavones are antioxidants [45], estrogen agonists/antagonists [46,47], topoisomerase inhibitors [48] and inhibitors of tyrosine kinases [49]. It has now been well recognized that isoflavone could target multiple pathways to induce apoptotic cell death. Apoptosis is a programed cell death, which occurs in cells during development and normal cellular processes but is suppressed in cancer. Multiple signaling pathways are impaired in tumor cells, leading to uncontrolled cell proliferation and resistance to apoptosis [50]. Isoflavones can activate apoptosis and enhance the anti-tumor effects of chemotherapeutic agents [51]. Different studies demonstrated that isoflavones could be useful either alone or in combination with conventional therapeutics for the prevention of tumor progression and/or treatment of the most human malignancies [51,52]. Isoflavones have been shown to reduce the risk of hormone-dependent tumors due to their potential estrogen-antagonistic effects [53]. The effects of isoflavones have been studied in different cancer cell lines, animal models and humans during clinical trials. Several clinical trials have been conducted to investigate the toxicity and effects of isoflavones in healthy men and women and in patients with prostate, breast, ovarian and colon cancer [54].

Isoflavones can bind to estrogen receptors (ER) and it provoked concerns that their use may lead to the development of estrogen-sensitive malignancies, especially in women at high risk or with breast cancer [55]. However, in vitro studies have shown that the proliferation of breast cancer is dependent on increased *α*-ER activity, and *β*-ER appears to inhibit *α*-ER-induced cancer cell proliferation [55]. Isoflavone derivatives generally induce receptor-dependent transcription and the induction is stronger with *β*-ER than with *α*-ER. The interactions of isoflavones with ER have been confirmed by studies in various cancer cell lines [56,57]. Reiter et al. conducted in an vitro study showing antiproliferative effects of red clover isoflavone extract in different human cancer cell lines: colon, prostate, breast, cervix, liver, pancreas, stomach, and ovaries [58]. In this study, the decreased rather than increased cell proliferation has been observed in the ER-positive MCF-7 breast cancer cells that grow under pre- and post-menopausal conditions [58]. Therefore, these results indicate that isoflavones do not pose a health risk.

In human studies, research has shown that soy isoflavones can improve prognosis in breast cancer patients. Chi et al. conducted analysis which revealed that isoflavone consumption (from soy food) may be a potential treatment option for ER negative, ER positive/progesterone receptor positive, and postmenopausal patients [59]. The results of the investigation performed by Guha et al. demonstrated that breast cancer recurrence was reduced with increasing amounts of daidzein consumption in a prospective cohort study of postmenopausal women who were treated at some point with tamoxifen [60]. It also was determined that protective effects of soy were stronger in postmenopausal women compered to premenopausal women [60].

Thus, isoflavones might be considered as potential bioactive compounds in the alternative therapies for the treatment and prevention of the hormone-related cancers.

### 4.2. Effects of Isoflavones in Metabolic Diseases

Isoflavones upregulate fatty acid metabolism, insulin sensitivity and adipocyte differentiation whereas they suppress type II diabetes and obesity [61]. Furthermore, isoflavones can modulate inflammation and NAD+ metabolism via endocrine and paracrine signaling pathways [62].

Genistein activated insulin secretion in pancreatic islets of neonate and adult mice [63], cAMP production and protein kinase A in pancreatic islet’s cell linings [64]. Furthermore, genistein was capable to decrease blood glucose levels [65] and to impair insulin binding to its receptor [66]. Genistein directly suppressed the insulin-induced glucose passage in 3T3-L1 adipocytes [67]. Moreover, genistein diminished insulin levels, the insulin resistant index and serum glucose, simultaneously decreasing transforming growth factor beta (TGF-*β*) concentration in ovariectomized rats [68]. Genistein suppressed the cAMP-activated cortisol synthesis in adult adrenocortical cell line H295 [69]. Genistein decreased glucocorticoid-induced obesity marker leptin production, and ERK1/2 phosphorylation upregulated adiponectin production [70]. Genistein was capable to increase adiponectin production, but to decrease leptin production in human synovial fibroblasts [71]. Isoflavones exerted a beneficial effect on lipid and glucose metabolism by activating PPAR in obese rats with type II diabetes [72]. PPAR*γ* activation is very important for the modulation of insulin sensitivity and blood glucose homeostasis [73]. Isoflavones could also activate receptors involved in fatty acid β-oxidation modulation—PPARα and PPAR*δ* [74]. Daidzein and genistein suppressed gluconeogenic enzyme activity in the liver and activated glucose-6-phosphate dehydrogenase and the malic enzyme, thus increasing hepatic glycogen amount, lowering blood glucose concentration and inhibiting the hepatic fatty acid *β*-oxidation in non-obese diabetic mice [75,76]. Genistein was found to increase the activities of catalase, superoxide dismutase, and glutathione peroxidase in livers of streptozotocin-induced diabetic rodents thus stimulating insulin sensitivity [77,78].

Epidemiological studies have shown that the increased intake of dietary soy isoflavones decrease diabetes cases and augment tissue sensitivity to insulin [79]. Short-term isoflavone-rich soy protein supplementation (30 g/day) improved glycemic control, reduced insulin resistance and lowered low-density lipoprotein cholesterol in postmenopausal women with type 2 diabetes mellitus in a double-blind, placebo-controlled cross-over study [80]. Additionally, higher intake of soy was associated with a reduced risk of type 2 diabetes mellitus in a prospective, population-based study of middle-aged Chinese women [81]. Thus, isoflavones might be beneficial in reducing risk of and/or alleviating the metabolic diseases.

### 4.3. Effects of Isoflavones in Cardiovascular Diseases

**Figure 5:**
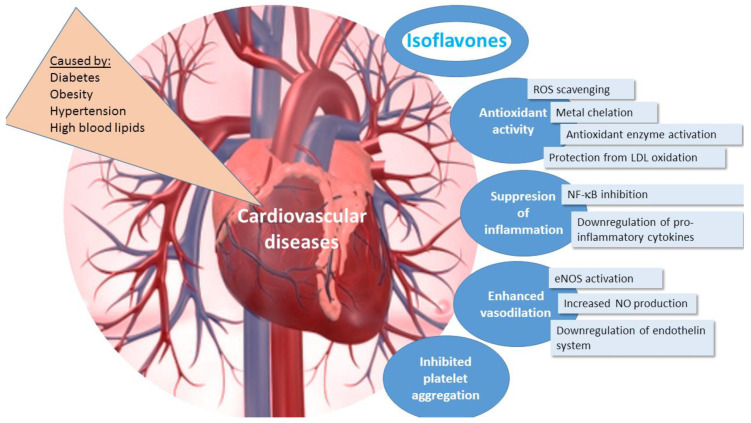
The effects of isoflavones in cardiovascular diseases. ROS—reactive oxygen species, LDL—low density lipoprotein, NF-κB—nuclear factor kappa B, eNOS—endothelial nitric oxide synthase, NO—nitric oxide.

Cardiovascular diseases: hypertension, dyslipidemia, coronary heart disease, and heart insufficiency are among the main causes of death in the world [82]. A soy-rich diet (at least 25 g of soy protein daily) has been shown to reduce the risk of cardiovascular diseases [83,84,85]. The beneficial activity of isoflavones for heart has been linked to their antioxidant, anti-inflammatory activities, enhanced vasodilation and inhibited platelet aggregation (Figure 5) thus preventing thrombosis and occlusion of blood vessels [12,86].

Nitric oxide produced by endothelial cells present in the inner surface of the blood vessels is a powerful vessel dilator, however this function is impaired under pathological conditions [87]. *β*-ER are present in the blood vessel endothelium and could be readily bind by isoflavones [88]. Isoflavones may upregulate endothelial nitric oxide synthase (eNOS) [89,90] and enhance NO production [91], thus reducing elevated blood pressure due to vasodilating activity [92]. 1–10 mM of genistein activated eNOS and increased NO production in human endothelial cells [93]. Genistein was also suggested to inhibit the NF-κB pathway [94]. Daidzein (40 mM) could suppress high-glucose–induced inducible nitric oxide synthase (iNOS), cyclooxygenase-2 (COX-2), and NF-κB expression in human endothelial cells, decreasing lipid peroxidation, reactive oxygen species (ROS) production and increasing NO levels [95]. The meta-analysis revealed that isoflavones could lower elevated blood pressure but had no effect on blood pressure under normal conditions [96]. Another meta-analysis demonstrated that isoflavones could increase flow-mediated dilation and improved endothelial function [97]. Isoflavones decreased systolic blood pressure thus protecting from cardiovascular disturbances [13,98,99]. Isoflavones might neutralize hydrogen peroxide in the cells due to their antioxidant activity thus modulating thromboxane production via the COX-1 pathway [100]. Isoflavones could also inhibit platelet-ADP collagen receptors, thus enhancing fibrinogen bin-ding to platelet surface receptors and leading to the decreased platelet aggregation and lowered probability of thrombosis [100,101,102].

Isoflavones-induced decrease in cholesterol could protect the endothelium of blood vessels [103]. Total decreases of 9.3% in total cholesterol, 12.9% in low-density lipoprotein and 10.5% in triglycerides have been reported in a meta-analysis of 38 research studies where on the average 50 g of soy protein was taken daily [104]. Similar decreases were observed in other studies demonstrating decreases in cholesterol, LDL and triglycerides [105,106,107,108,109]. However, the data are contradictory, and several meta-analysis studies reported no isoflavone-induced changes in serum lipid profile [84,110]. Thus, the beneficial effects of isoflavones in the cardiovascular system might be related to their ability to protect against LDL oxidation rather than to the direct modulation of their concentrations in the blood [111,112,113].

Thus, isoflavones are promising cardioprotective compounds although more detailed studies are required to clearly confirm their beneficial effects in humans.

### 4.4. Effects of Isoflavones in Neurodegenerative Diseases

Estrogen receptors have been found in the central nervous system, suggesting a role for estrogens in the functions of learning and/or memory. Consequently, isoflavones may exert beneficial effects on the cognitive function, because they structurally resemble 17*β*-estradiol [114]. In the study of White et al. it was reported that estrogen may play a role in repairing age-related brain tissue degradation, particularly in the structures linked to the memory and executive function such as the neocortex and hippocampus [115]. Pathological cognitive aging, such as Alzheimer’s disease share many risk factors with cardiovascular disease, probably allowing phytoestrogens to exert protection on the brain through these mechanisms [116].

Cognitive decline is related to the aging processes [117]. In women cognitive decline is linked to the loss of estrogen during and after the menopause [118] and its impaired modulation of cellular functions [119]. Estradiol activity is important in the formation of dendritic spines and synapses in brain [120]. The dietary intake of soy isoflavones have been shown to exert neuroprotective activity in mice [121] and rats [122,123], although supplementation with high doses (20 mg/day) were cytotoxic due to apoptotic activity and increased levels of the marker of neuronal damage—lactate dehydrogenase [124]. Genistein was neuroprotective and had less adverse effects compared to the synthetic estradiol in the cerebral cortex of elderly rats model [125]. Daidzein inhibited apoptosis and could reverse toxic effects of glutamate in neuronal cells acting via G protein-coupled estrogen receptor 1 (GPER-1) and ER*α*, whereas genistein had an influence on the development of hypothalamic neurons, by increased neuritic arborization through the ER*α*, ER*β* and GPER1 in vitro [126]. Genistein could prevent inflammation and alleviate Alzheimer’s disease in preclinical models. Genistein increased expression levels of PPAR*γ* thus preventing inflammation in cultured astrocytes [127]. Activated PPAR*γ* decreased the expression levels of NF-κB [128]. The expression levels of pro-inflammatory cytokines such as iNOS, COX-2, TNF-*α*, IL-1*β*, IL-6 were decreased in astrocytic glial cells [127,129], in hippocampal neurons [130], in cortical neurons [131] treated with genistein in vitro. Thus, isoflavones could protect from neuronal inflammation.

Henderson et al. conducted double-blind soy isoflavones trial with 350 healthy postmenopausal women [132]. Women in the study received daily 25 g of isoflavone-rich soy protein (91 mg of aglycone weight of isoflavones: 52 mg of genistein, 36 mg of daidzein, and 3 mg glycitein) or milk protein matched placebo. After the study authors concluded that long-term dietary soy isoflavone supplementation in a dose comparable to that of traditional Asian diets has no effect on global cognition but may improve visual memory [132]. In another study, sixty-five men and women over the age of 60 were treated with 100 mg/day soy isoflavones, or matching placebo capsules for six months [133]. The study was conducted to examine the potential cognitive benefits of soy isoflavones in patients with Alzheimer’s disease (52.3% women, and 47.7% were apolipoprotein E *ε*4 positive). The study demonstrated that the treatment with soy isoflavones had no significant effects on the cognition in older men and women with Alzheimer’s disease [133]. The study of Kritz-Silverstein et al. with postmenopausal women showed more promising results [134]. The research was conducted for 6 months and it was double-blind, randomized and placebo-controlled. A total of 56 women were randomized into two groups (placebo group and active treatment group). Women randomized to the active treatment group (n = 27) took two pills per day, each containing 55 mg of soy-extracted isoflavones (110 mg total isoflavones per day). Women assigned to the placebo group (n = 26) took two identical-appearing pills per day containing inert ingredients. The results suggested, that isoflavone supplementation had a favorable effect on cognitive function, particularly verbal memory, in postmenopausal women [135].

However, despite that the neuroprotective effects of isoflavones have been observed in vitro in various cell cultures and in animal models, the results from the clinical trials in humans were contradictory [136] and more studies could be recommended to be able to draw the conclusion about the beneficial effects of isoflavones in neurodegenerative diseases [3]. Moreover, the use of high-throughput screening [137] and the computer-aided drug design [138] could also be valuable tools for the investigation of potential isoflavone-interacting proteins and their active sites. Furthermore, the recent discovery of the glymphatic system, which promotes the efficient elimination of soluble proteins and metabolites from the central nervous system [139,140] and has been suggested to have a role in neurodegenerative diseases [141], as well as the participation of water channel aquaporin 4 in the regulation of the glymphatic system [140,142] could be of interest as possible targets of isoflavones to be investigated in the future [143].

### 4.5. Effects of Isoflavones in Rheumatoid Arthritis

Rheumatoid arthritis is a chronic autoimmune disease usually diagnosed for people around 60 years old that affects more women than men [144]. Inflammation impairs flexible joints and tissues causing joint swelling, stiffness and pain during the development of the disease [145]. The cellular mechanisms involved in rheumatoid arthritis are linked to the function of monocytes, macrophages and T cells, the suppressed immune response being a marker of the disease progression [146]. The strategy used for the inflammation treatment usually involves the neutralization of pro-inflammatory cytokines [147]. Isoflavones have been shown to suppress inflammation via interaction with various molecular targets [12,24,86,148].

The studies of Verdrengh et al. revealed that subcutaneous injection of genistein (30 mg/kg body weight) suppressed the inflammation in collagen-induced arthritis model in rats modulating the functions of granulocytes, monocytes, and lymphocytes [35]. Furthermore, genistein exerted the anti-leptin activity inhibiting inflammation in rheumatic diseases model [149]. Additionally, the elevated levels of IL-1*β* or TNF-*α*-activated MMP-9 and MMP-2 in rheumatoid synoviocytes were significantly reduced by genistein treatment [150]. Moreover, genistein decreased a Th1-predominant immune response in collagen-induced rheumatoid arthritis model in rats via suppression of the secretion of interferon-gamma (IFN-*α*) and IL-4 [151]. In the study of Cheng et al., genistein suppressed IL-6-induced vascular endothelial growth factor (VEGF) expression and angiogenesis partially through the Janus kinase 2 (JAK2)/STAT3 pathway in rheumatoid arthritis model in MH7A cells in vitro [152]. 20 mg/kg genistein or daidzein gavaged to the female DBA1/J mice in collagen induced arthritis model exerted protective effects by increasing IgG glycosylation leading to amelioration of inflammation and inhibiting the NF-κB pathway and NFATc1/c-Fos thus decreasing the activity of osteoclasts [153]. In the study of Hu et al., 5 mg/kg of genistein was administered for 12 days to DBA/1 mice subjected to collagen-induced arthritis [154]. The results revealed that genistein suppressed the expressions of IL-1*β*, IL-6 and TNF-*α* in the serum and decreased VEGF expression, inhibited angiogenesis in the synovial tissue [154]. In an experimental model of collagen-induced rheumatoid arthritis in Wistar albino rats, a suspension of daidzein (20 mg/kg body weight) was orally administrated twice daily for 21 days, resulting in the decreased inflammatory markers and arthritis scoring [155].

Activation of osteoclasts and overexpression of cytokine-induced destructive enzymes of matrix metalloproteinase (MMP) family are linked to the collagen degradation and bone erosion which further causes joint destruction in rheumatoid arthritis [148,156]. A total of 50 μM genistein was able to decrease the expression of most of MMPs in MCF-7 and PC3 cells [157] and upregulate the expression of osteoprotegerin [158]. Furthermore, genistein (0.1 to 10 μM) suppressed osteoclastogenesis and activated apoptosis of mature osteoclasts in mouse marrow culture [159]. In addition, 10 μM of genistein could stimulate differentiation and mineralization of osteoblasts and activated protein synthesis in osteoblasts in vitro [160]. The effects of equol administration were investigated on the inflammatory response and bone erosion in mice with collagen-induced arthritis [161]. The results showed the decreased severity of arthritis symptoms [161].

Thus, the in vitro and animal studies of the effects of isoflavones in rheumatoid arthritis support these compounds as potential natural remedies that could be used as the complementary treatment in this disease.

### 4.6. Effects of Isoflavones in Other Degenerative Diseases

Osteoporosis is a degenerative skeletal disease characterized by deteriorating bone microarchitecture, low bone mineral density and greater bone resorption than bone formation [162]. Decreased estrogen levels in post-menopausal women are a critical risk factor for osteoporosis development among older female adults. However, it is well established in the literature that hormone replacement therapy significantly increases the risk of both fatal and non-fatal cardiovascular disease as well as breast cancer [163,164].

Atkinson et al. conducted randomized, double-blind, and placebo-controlled study, involving 205 women [165]. The results showed that women taking red clover isoflavonoids (43.5 mg/d) for 12 months had lower reductions in lumbar spine mineral content and bone density compared with the placebo-controlled group. An increase in the markers of bone formation was also observed [165]. In other randomized, double blind clinical trial with 46 postmenopausal women the effects of phytoestrogens on lipid and bone metabolism were observed [166]. Women received a randomized dose of isoflavonoids of 28.5, 57, or 85.5 mg/d for 6 weeks. After 6 weeks of isoflavone therapy, an increase in bone density was observed compared to the control. Subjects treated with 57 mg/d of isoflavones had an increase in bone density of 4.1%, those treated with 85.5 mg/d had a 3% increase in bone density, and those treated with 28.5 mg/d had a negligible change in bone density [166].

Isoflavones have been shown to reduce menopausal symptoms like hot flashes, and this effect is linked to the estrogenic activity of isoflavones. However, in the study of Nissan et al. it was discovered, that isoflavones can bind to *µ*- and *δ*-opiate receptors. This mechanism could help explain the positive effects of isoflavones on menopausal symptoms as the opioid system regulates temperature, mood and hormone levels [167].

Isoflavone daidzein has been shown to stimulate the hyaluronic acid production and to protect the skin from oxidative damages induced by ultraviolet radiation following topical application. Therefore, daidzein seems to be a promising agent for skin aging prevention, especially for postmenopausal women [168].

Thus, isoflavones might serve as natural remedies in alleviating menopause-related symptoms without the risk of side effects that are common during the use of synthetic estrogen as a hormone replacement therapy.

## 5. Conclusions and Future Perspectives

Isoflavones are potent phytoestrogens and antioxidants capable to protect cells and restore their normal functions in many pathological conditions. Isoflavones decrease inflammation, suppress oncogenic processes, and exert beneficial effects during aging and estrogen depletion. Although more human trials would be beneficial to support the use of isoflavones in alternative therapies, due to their pleiotropic activities isoflavones might be considered as natural alternatives protecting from the degenerative diseases.

## Figures and Tables

**Table 1:** Main isoflavone aglycones (genistein, daidzein, glycitein) and their isoforms.

*Compound*	*R*_1_	*R*_2_	*R*_3_	*R*_4_
Genistein	H	H	OH	H
Genistin	C_6_O_5_H_11_	H	OH	H
Acetyl-genistin	C_6_O_5_H_11_ + COCH_3_	H	OH	H
Malonyl-genistin	C_6_O_5_H_11_ + COCH_2_COOH	H	OH	H
Daidzein	H	H	H	H
Daidzin	C_6_O_5_H_11_	H	H	H
Acetyl-daidzin	C_6_O_5_H_11_ + COCH_3_	H	H	H
Malonyl-daidzin	C_6_O_5_H_11_ + COCH_2_COOH	H	H	H
Glycitein	H	OCH_3_	H	H
Glycitin	C_6_O_5_H_11_	OCH_3_	H	H
Acetyl-glycitin	C_6_O_5_H_11_ + COCH_3_	OCH_3_	H	H
Malonyl-glycitin	C_6_O_5_H_11_ + COCH_2_COOH	OCH_3_	H	H

## Data Availability

Not applicable.
